# USMRI Features and Clinical Data-Based Model for Predicting the Degree of Placenta Accreta Spectrum Disorders and Developing Prediction Models

**DOI:** 10.1155/2022/9527412

**Published:** 2022-01-31

**Authors:** Peng An, Junyan Zhang, Feng Yang, Zhongqiu Wang, Yan Hu, Xiumei Li

**Affiliations:** ^1^Department of Radiology, Xiangyang No. 1 People's Hospital, Hubei University of Medicine, Xiangyang 441000, China; ^2^Department of Radiology, The Affiliated Hospital of Nanjing University of Chinese Medicine, Jiangsu Province Hospital of Chinese Medicine, The First Clinical Medical College, 155 Hanzhong Road, Nanjing 210029, Jiangsu Province, China; ^3^Department of Pharmacy and Laboratory, Xiangyang No. 1 People's Hospital, Hubei University of Medicine, Xiangyang 441000, China

## Abstract

**Aim:**

This study aimed to investigate the ability of ultrasound/magnetic resonance imaging (MRI) signature and clinical data-based model for preoperatively predicting the degree of placenta accreta spectrum disorders and develop combined prediction models.

**Methods:**

The clinicopathological characteristics, prenatal ultrasound images, and MRI features of 132 pregnant women with placenta accreta spectrum disorders at Xiangyang No. 1 People's Hospital were retrospectively reviewed from January 2016 to December 2020. In the training set of 99 patients, the ultrasound/MRI features model, clinical characteristics model, and combined model were developed by multivariate logistic regression analysis to predict the degree of placenta accreta spectrum disorders. The prediction performance of different models was compared using the Delong test. The developed models were validated by assessing their prediction performance in a test set of 33 patients.

**Results:**

The multivariate logistic regression analysis identified history of abortion, history of endometrial injury, and blurred boundary between the placenta and the myometrium/between the uterine serosa and the bladder to construct a combined model for predicting the degree of placenta accreta spectrum disorders (area under the curve (AUC) = 0.931; 95% confidence interval (CI): 0.882–0.980). The AUC of the clinical characteristics model and ultrasound/MRI features model was 0.858 (95% CI 0.794–0.921) and 0.709 (95% CI 0.624–0.798), respectively. The AUC of the combined model was significantly higher than that of the ultrasound/MRI features model (*P* < 0.001) or clinical characteristics model (*P* < 0.0015) in the training set. In the test set, the combined model also showed higher prediction performance.

**Conclusions:**

Ultrasound/MRI-based signature is a powerful predictor for the degree of placenta accreta spectrum disorders in an early stage. A combined model (constructed with history of abortion, history of endometrial injury, and blurred boundary between the placenta and the myometrium/between the uterine serosa and the bladder) can improve the accuracy for predicting the degree of placenta accreta spectrum disorders in an early stage.

## 1. Introduction

The first case of placenta accreta listed on PubMed was reported in 1927 by Dr D. S. Forster. Hertig and Jauniaux reported placenta accreta spectrum disorders and confirmed that this condition could lead to postpartum hemorrhage, hysterectomy, and death of parturients [1–3]. Placenta accreta spectrum disorders are divided into placenta accreta (PA), placenta increta (PI), and placenta percreta (PP) depending on the depth of attachment and invasion into the myometrium of the uterus [3, 4]. PA refers to the condition in which the placental villi penetrate through the decidua and attach to the myometrium. PI is the condition in which the placental villi invade the myometrium. PP is the condition in which the placental villi penetrate all three layers of the uterus, reaching the serosal layer or even invading the adjacent organs. PP is a more critical condition that may lead to the death of both the mother and the baby. According to the statistics, the incidence of PA spectrum disorders has increased in recent years and reached 0.18% in the USA and 0.47% in China [5, 6].

PA spectrum disorders are usually diagnosed antepartum by ultrasound (US) and magnetic resonance imaging (MRI). The diagnostic sensitivity of US for PA spectrum disorders is 78%, and the specificity is 78.6% [4, 7]. However, US may not diagnose all cases of PA spectrum disorders due to sound attenuation and interference from the surrounding tissues. MRI has the advantages of noninvasiveness, multiplanar reformation, high tissue resolution, and high sensitivity to blood flows. The diagnostic sensitivity and specificity are reported to be 88% and 92%, respectively [4, 6, 8]. Therefore, MRI is more likely to accurately depict the depth of implantation and parauterine and cervical involvement. The MRI findings can be used to guide surgical treatment and reduce postpartum hemorrhage and other complications [9, 10]. However, MRI scanning time is long, and the cost is high, and hence, it cannot be widely carried out. Previous studies often used US imaging features to predict the degree of placenta accreta spectrum disorders, but the accuracy was quite low and the false-positive rate was high. Therefore, subsequent MRI is beneficial after US detects some suspicious features. In this study, the US/MRI imaging model, clinical characteristics model, and combined model were established. The significant clinical value of the combined model in diagnosing the degree of PA spectrum disorders was revealed by comparing receiver operating characteristic curves. In addition, 99 patients were selected to establish a training set and modeled. The remaining 33 patients in the test set were verified, and good results were obtained. It is reported as follows.

## 2. Materials and Methods

**Figure 1:**
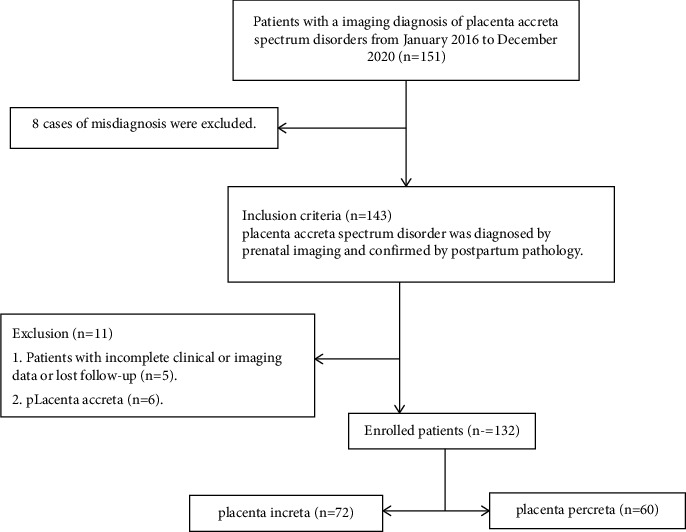
Flow chart showing inclusion and exclusion of subjects in this study.

Clinical data: the clinical, pathological, and imaging data were collected from 132 pregnant women with PA spectrum disorders from January 2016 to December 2020. They were confirmed as PI or PP by postoperative pathology and included in our study (72 in the PI group and 60 in the PP group) (Figure 1). Data collection: the demographic features included age (18–48 years, with an average of 30.11 ± 7.13), pregnancy times, history of abortion, history of cesarean section, history of placenta previa, history of PA spectrum disorders, and history of endometrial injury, uterine fibroid, hysteroscopy, and adnexal cyst. In this study, we obtained the informed consent of all patients. The study was approved by the Human Ethics Committee of Xiangyang No. 1 People's Hospital affiliated to the Hubei University of Medicine (issue no. S109 [2016]).

### 2.1. Diagnostic Methods

#### 2.1.1. MRI Examination

A Philips Achieva Nova Dual 1.5 T MRI scanner (Philips Medical Systems, Best, Netherlands) with a 6-channel phased-array body coil was used for the examination. The pregnant women were placed in a supine or left lateral position. The middle and lower abdomen and the pelvic cavity were first located. Then, the transverse, sagittal, and coronal T2WI and sagittal T1WI images were acquired for the uterus. The scan ranged from about 2 cm above the fundus of the uterus to the pubic symphysis. T2WI was acquired by the single-shot fast spin echo (SSTSE) and balanced fast field echo (balance-FFE). The SSTSE parameters were as follows: TR, 7500 ms; TE, 100 ms; flip angle, 90°; matrix, 300 × 00; slice thickness, 5 mm; and field of view (FOV), 26 × 26 cm. The balance-FFE parameters (in breath-holding) were as follows: TR, 3.60 ms; TE, 1.8 ms; flip angle, 90°; and slice thickness, 8 mm. T1WI was acquired by fast spin echo in breath-holding: TR, 800 ms; TE, 15 ms; matrix, 256 × 256; slice thickness, 2–5 mm; and FOV, 26 × 26 cm [4, 9].

#### 2.1.2. US Examination

A Mindray R7 scanner(Mindray R7, Shenzhen Mindray Bio-Medical Electronics Co., Ltd., Guangdong, China) and a GE E8 US system (Voluson Expert E8, General Electric, Kretz Ultrasound, Zipf, Austria) with a RAB4-8L transducer and an ordinary array transducer were used for the examination. The scan was performed according to the International Society of Ultrasound in Obstetrics & Gynecology (ISUOG) diagnostic guidelines for PA spectrum disorders. The abdominal and endoluminal convex array probe frequency was 3.0–5.0 MHz or 4.0–8.0 MHz. The middle and late pregnancy abdominal modes of obstetric US were selected. Pregnant women required moderate bladder filling and were placed in the supine or lateral position. After the routine examination of the fetus, the placenta was carefully checked, and the thickness of the placenta, placenta previa, disappearance of retroplacental space, thinning or disappearance of retroplacental myometrium, irregular uterine bladder boundary, aberrant abundant blood stream signals behind the placenta, and so forth were recorded [6].

#### 2.1.3. Imaging Findings

Crucial MRI and US characteristics included complete placenta previa, blurred boundary between the placenta and the myometrium, blurred boundary between the uterine serosa and the bladder, continuity of echoic line in the myometrium, abnormal retroplacental blood flow signals, signs of bladder involvement, and signs of cervical involvement [4, 11, 12]. Two radiologists (An peng and Yang feng, with 10 and 7 years of experience in abdominal-obstetric radiology, respectively) made imaging diagnostic results independently and came to an agreement.

#### 2.1.4. Pathological Diagnosis

PA spectrum disorders were diagnosed with the following microscopic findings: the placental villi and decidua were maldeveloped or no decidua was present. The villi came into direct contact with the myometrium or penetrated deep into the myometrium [7, 13, 14].

### 2.2. Statistical Methods

All statistical of mechanical analyses were performed using the SPSS 22.0 software (IBM, Armonk, NY, USA). Measurement data obeying a normal distribution were represented by *X* ± *s*, and intergroup comparisons were performed by using the independent samples *t*-test or by the *χ*2 test or Fisher's exact test. Multiple logistics regression analysis was carried out. The odds ratio (OR) and the 95% confidence interval (95% CI) were calculated, based on which the correlation between the risk factors and the severity of placenta accreta spectrum disorders was assessed. *P* < 0.05 was taken to indicate a significant difference. Receiver operating characteristic (ROC) curves were generated, and the area under the curve (AUC) was used to evaluate the accuracy of US/MRI features model, clinical characteristics model, and combined model in predicting the degree of placenta accreta spectrum disorders. Then, the developed models were validated by assessing their prediction performance in test set. Comparisons between three models were performed using the Delong test in the training and test sets. Higher prediction accuracy presented with a larger AUC and a *p* value <0.05 (two-tailed) indicated statistical significance. Decision curve analysis (DCA) of training and test sets were conducted to determine the clinical usefulness by quantifying the net benefits at different threshold probabilities in the models. Survival curves were drawn by using the Kaplan–Meier method, and differences of survival rates were compared with the log rank, Breslow, and Tarone-Ware test. Other statistical analyses were done using R software (R Foundation for Statistical Computing, version 3.5.8; https://www.r-project.org/) [15].

## 3. Results

### 3.1. Patient Medical History Characteristics

The PI and PP groups differed significantly in terms of pregnancy times, age, history of cesarean section, history of abortion, and history of endometrial injury (all *P* < 0.05) (Table 1).

Among all recruited patients, 98 had a history of abortion and 58 had a history of multiple abortions (≥2 times). The incidence of PP increased with the increase in the number of artificial abortions (Table 2).

### 3.2. Development and Performance of Prediction Models

The univariate logistic regression analysis was performed for the potential risk factors and the US and MRI characteristics. The risk factors for PA spectrum disorders included pregnancy times, age, history of cesarean section, history of abortion, history of endometrial injury, complete placenta previa, blurred boundary between the uterine serosa and the bladder, and blurred boundary between the placenta and the myometrium (all *P* < 0.05) (Tables 3 and 4).

The multivariate logistic regression analysis based on ultrasound/MRI imaging features and clinical characteristics identified history of abortion, history of endometrial injury, blurred boundary between the placenta and myometrium, and blurred boundary between the uterine serosa and bladder to construct combined model for predicting PP (AUC = 0.931; 95% CI: 0.882–0.980) (Table 5).

**Figure 2:**
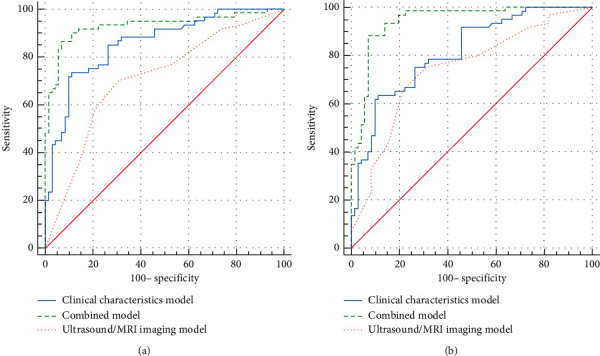
Delong nonparametric approach. AUC estimates for predicting early placenta accreta spectrum disorders were compared between different prediction models in the training set (a) and test set (b).

AUC estimates were compared between prediction models by using the Delong nonparametric approach in training and test sets. In the training set, the AUC of the combined model was significantly higher than that of the ultrasound/MRI imaging features model (*p* < 0.001) and clinical characteristics model (*p*=0.0015). In the test set, the combined model yielded the excellent AUC (0.944; 95% CI: 0.904–0.983) (Figure 2).

**Figure 3:**
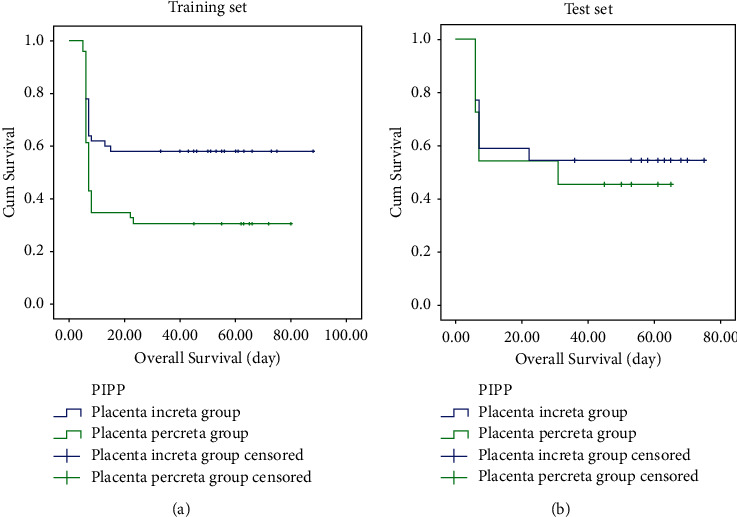
Kaplan–Meier survival curves of placenta accreta spectrum disorders patients with and without PP in the training set (a) and test set (b).

The overall fetal survival or nonhysterectomy in placenta accreta spectrum disorders patients with PP was significantly poorer than those without PP in both training set (*p* < 0.001) and test set (*p* < 0.001) (Figure 3).

### 3.3. Clinical Application

**Figure 4:**
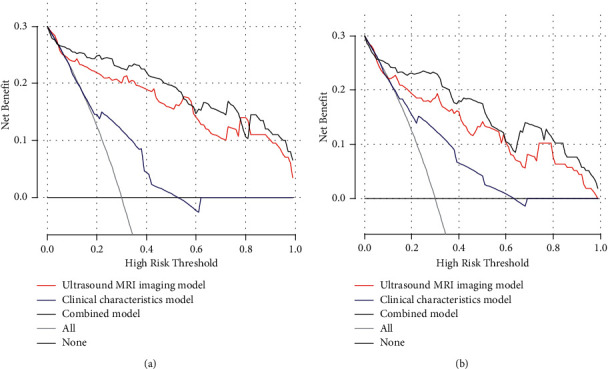
Decision curve analysis in the training set (a) and test set (b). Decision curves of the ultrasound/MRI imaging model, clinical characteristics model, and combined model.

DCA in the training and test sets for the ultrasound/MRI imaging features model, clinical characteristics model, and combined model was performed. The highest curve (representing the combined model) at most of the given threshold probability is the optimal decision-making strategy to maximize the net benefit compared with other two models (Figure 4).

## 4. Discussion

Placenta accreta spectrum disorders are a primary cause of postpartum hemorrhage, uterine perforation, maternal hemorrhagic shock, infectious acute abdomen, perinatal emergency partial hysterectomy or adnex removal, and maternal or fetal death [16, 17]. The maternal mortality caused by PA spectrum disorder/especially PP complicated with massive hemorrhage after a cesarean section has gradually increased with the opening of the second child policy and the increase in the number of elderly and multiple pregnant women [17, 18]. In recent years, the pathogenesis of penetrating invasive placenta has attracted extensive attention. With the continuous progress of research, scholars generally believe that the occurrence of penetrating invasive placenta is related to the lack of placental decidua basalis, the strong invasion of trophoblasts, and the disorder of uterine spiral artery reperfusion [18, 19]. Placenta accreta spectrum disorders are divided into PA, PI, and PP based on the relationship between placental villi and myometrium. The prenatal diagnosis of PA is difficult, but the PA-related harm is relatively small, with no clear indication of clinical treatment. Therefore, it was not included in this study. PI needs short-term follow-up or clinical observation, which mainly affects the surgical style or increases the scope of myometrium resection during operation or postoperative repair, but all these depend on the accurate evaluation of prenatal imaging. PP is a crisis event in clinical decision-making. For patients with PP with different gestational ages and stages of labor, a detailed operation plan should be formulated according to the US/MRI results to try to reduce postpartum hemorrhage, amniotic fluid embolism, and peripheral tissue resection; preserve the uterus and uterine appendages; and prevent uterine perforation and abdominal infection. It is precisely because PP increases maternal mortality and doctor-patient disputes. Therefore, predicting the severity of PA spectrum disorders is important [20].

In the present study, a retrospective analysis in the clinical characteristics model was performed to identify the risk factors for PP in PA spectrum disorders. The number of previously received abortions and history of endometrial injury were independent risk factors for PP. The incidence of PP in pregnant women with a history of abortion was significantly higher than that in those without a history of abortion (57 vs. 3). Therefore, the history of abortion is an important factor for predicting PP. The incidence of PP also increased with the number of previously received times of abortions. Moreover, the history of abortion with endometrial injury was associated with a higher possibility of PP than a history of abortion without endometrial injury (41.66% (55/132) vs. 3.78% (5/132)). The incidence increased to 43.18% (57/132) among those with a history of ≥1 abortions. As the number of previously received abortions increased, the OR for predicting PP increased (1 time: OR 5.564, 95% CI: 1.440–21.495; 2 times: OR 2.786, 95% CI: 1.048–7.045; ≥3 times: OR 5.556, 95% CI: 1.366–22.591). Furthermore, the age, pregnancy times, and history of cesarean section were also significant risk factors for PP (all *P* < 0.05). Therefore, these valuable data should be closely followed by obstetricians and gynecologists. Moreover, inquiring about a detailed medical history is essential to diagnose PP.

Prenatal US can be used to assess the depth of PA spectrum disorders with the following indicators: local or scattered vacuolated blood flows in the placenta, sinusoid formation, formation of blood vessels in the uterovesical space, and retroplacental venous plexus. The 3D US is also applicable to PA spectrum disorders. So far, no consensus has been reached on the US indicators and diagnostic criteria for the degree of PA spectrum disorders. The US characteristics considered in the present study also included, apart from the aforementioned four, continuity of the echoic line in the myometrium and signs of bladder and cervical involvement. These findings could also assist with the diagnosis of PI and PP. We carried out a comprehensive evaluation, which also included the age, pregnancy times, and history of uterine cavity surgery, to improve the diagnostic sensitivity and specificity of US for PP. The diagnostic value of each of the US indicators was assessed accordingly using these factors. The diagnostic accuracy was considerably improved for PP [21, 22].

US scanners are susceptible to the limitations of US itself, which makes missed diagnosis inevitable. In contrast, MRI is free from the interferences from gases and surrounding tissues and therefore is highly suitable for the diagnosis of PP. The primary features of PP are identified as follows: multiple local thickening, bulging, and gathering of the placenta, the maternal side of the placenta concaving outward in a hump-like pattern and having a blurred boundary, and disruption of the hyposignal line adjacent to the myometrium. The placental tissues penetrate the uterine wall and invade the parauterine tissues, with the discovery of placental tissues outside the uterus. A hyposignal band and abnormal blood vessels in the placenta may be present, with a significantly uneven signal pattern and uterine bulging where the placenta attaches [23, 24]. All of the 132 pregnant women underwent US and MRI examinations. Among them, 95 patients (71.96%, 95/132) were found with PA spectrum disorders using US. Moreover, the blurred boundary between the uterine serosa and the bladder was more significantly correlated with PP (all *P* < 0.05). Furthermore, 115 patients were found with PA spectrum disorders using MRI, which revealed that complete placenta previa and blurred boundary between the uterine serosa and the bladder/between the placenta and the myometrium were more significantly correlated with PP (all *P* < 0.05). MRI offers an excellent complement to prenatal US. MRI is highly necessary if PA spectrum disorders are suspected by ultrasound [9, 25, 26].

Usually pregnant women are not asked to undergo MRI directly, but when US finds some abnormalities such as placenta thickening, complete placenta previa, and unclear boundary between placenta and uterus, we strongly recommend pregnant women to have an MRI examination to exclude PP. In addition, in the process of diagnosis of PA spectrum disorders, abnormal signs are found using US and can be further verified using MRI, which avoids misdiagnosis. Therefore, the US and MRI features of PA have a strong complementarity, and it is of great significance to combine the two in predicting the degree of PA spectrum disorders [27].

### 4.1. Limitations

The limitations of this study were as follows. First, the sample size was insufficient, with fewer patients in the test set; they all came from the same hospital. A multicenter study should be conducted to validate the results in the future. Second, although a dynamic contrast-enhanced MRI scan can clearly show the boundary between the placenta and the myometrium, the use of gadolinium chelate during pregnancy causes potential harm to the fetus. Therefore, this study did not explore the value of contrast-enhanced MRI.

## 5. Conclusions

Taken together, accurately predicting the degree of PA spectrum disorders using the combined model is valuable for reducing the mortality of parturients. The prenatal diagnosis and classification of PA spectrum disorders using US and MRI are the first step to build the predictive model for the severity of PA spectrum disorders and hence to predict the prognosis.

## Figures and Tables

**Table 1:** Comparison of risk factors between the PI group and PP group.

Group	*N*	Age (years)		Pregnancy times [*M*(*P*_25_ − *P*_75_)]	History of abortion		History of cesarean section		History of placenta previa		History of placenta accreta spectrum disorders		Uterine fibroid		Hysteroscopy		Adnexal cyst		History of endometrial injury	
		<32	≥32		YES	NO	YES	NO	YES	NO	YES	NO	YES	NO	YES	NO	YES	NO	YES	NO
PI	72	50	22	2 (0–3)	41	31	52	20	56	16	19	53	31	41	22	50	60	12	26	46
PP	60	11	49	3 (1–4)	57	3	57	3	54	6	16	44	27	33	19	41	52	8	51	9
*X* ^2^/*F*	34.39			2.13	24.78		10.27		3.52		0.0013		0.05		0.0188		0.2828		32.18	
*P* value	*P* < 0.05			*P* < 0.05	*P* < 0.05		*P* < 0.05		*P* > 0.05		*P* > 0.05		*P* > 0.05		*P* > 0.05		*P* > 0.05		*P* < 0.05	

**Table 2:** Correlation analysis between the number of previous abortions and the degree of placenta implantation.

Category	PI(72)	PP(60)		
	N	N	OR value	95% CI
0 time	31	3	—	—
1 time	26	14	5.564	1.440–21.495
2 times	12	18	2.786	1.048–7.405
≥3 times	3	25	5.556	1.366–22.591

**Table 3:** Logistic regression analysis for predicting early placenta accreta spectrum disorders based on the clinical characteristics model (clinical model).

Clinical characteristics model	Univariate analysis		Multivariate analysis	
	*P*	Hazard ratio	*P*	Hazard ratio
Pregnancy times	<0.05 ^*∗*^	2.241 (1.549–3.242)		
Age	<0.05 ^*∗*^	1.113 (1.054–1.176)		
History of cesarean section	<0.05 ^*∗*^	2.391 (1.648–3.471)		
History of abortion	<0.05 ^*∗*^	2.557 (1.802–3.627)	<0.05 ^*∗*^	3.208 (2.004–5.134)
History of endometrial injury	<0.05 ^*∗*^	3.289 (1.959–5.521)	0.008 ^*∗*^	2.574 (1.282–5.168)
History of hypertension	0.975	0.989 (0.498–1.963)		
Smoking history	0.846	0.933 (0.465–1.875)		
Drinking history	0.748	0.893 (0.447–1.784)		
Diabetes history	0.655	1.532 (1.652–4.617)		

^*∗*^ *p* < 0.05.

**Table 4:** Logistic regression analysis for predicting early placenta accreta spectrum disorders based on the ultrasound/MRI imaging model.

Ultrasound/MRI imaging model	Univariate analysis		Multivariate analysis	
	*P*	Hazard ratio	*P*	Hazard ratio
Marginal placenta previa	0.561	0.791(0.359–1.743)		
Complete placenta previa	0.029 ^*∗*^	2.195(1.085–4.440)	0.016 ^*∗*^	2.552(1.191–5.470)
Blurred boundary between the placenta and myometrium	0.008 ^*∗*^	2.604(1.280–5.296)	0.033 ^*∗*^	2.241(1.066–4.712)
Placental blood pool	0.973	1.012(0.493–2.077)		
Continuity of the myometrium	0.097	1.769(0.902–3.471)		
Blurred boundary between the uterine serosa and bladder	0.013 ^*∗*^	2.456(1.212–4.978)	0.013 ^*∗*^	2.643(1.232–5.668)
Signs of bladder involvement	0.192	1.619(0.784–3.341)		
Signs of cervical involvement	0.739	0.885(0.431–1.819)		
Abnormal retroplacental blood flow signals	0.924	0.967(0.486–1.924)		
Local thickening of placenta	0.501	0.788(0.394–1.577)		
Placental signal disorder	0.924	1.034(0.520–2.056)		
Placental protrusion	0.386	0.735(0.366–1.475)		

^*∗*^ *p* < 0.05.

**Table 5:** Logistic regression analysis for predicting early placenta accreta spectrum disorders based on ultrasound/MRI imaging characteristics and clinical characteristics (combined model).

Combined model	Univariate analysis		Multivariate analysis	
	*P*	Hazard ratio	*P*	Hazard ratio
Pregnancy times	<0.05 ^*∗*^	2.241 (1.549–3.242)		
Age	<0.05 ^*∗*^	1.113 (1.054–1.176)		
History of cesarean section	<0.05 ^*∗*^	2.391 (1.648–3.471)		
History of abortion	<0.05 ^*∗*^	2.557 (1.802–3.627)	<0.05 ^*∗*^	3.587 (2.077–6.195)
History of endometrial injury	<0.05 ^*∗*^	3.289 (1.959–5.521)	0.023 ^*∗*^	2.371 (1.125–4.994)
Complete placenta previa	0.029 ^*∗*^	2.195 (1.085–4.440)		
Blurred boundary between the placenta and myometrium	0.008 ^*∗*^	2.604 (1.280–5.296)	0.006 ^*∗*^	4.225 (0.859–3.594)
Blurred boundary between the uterine serosa and bladder	0.013 ^*∗*^	2.456 (1.212–4.978)	0.009 ^*∗*^	4.857 (1.490–15.827)

^*∗*^ *p* < 0.05.

## Data Availability

All data generated or analysed during this study are included in this published article.
